# Advanced squamous-cell carcinoma of the lower lip

**DOI:** 10.11604/pamj.2018.30.148.14352

**Published:** 2018-06-20

**Authors:** Fred Bernardes Filho, Priscila Vinhal Grupioni

**Affiliations:** 1 Dermatology Division, Department of Medical Clinics, Ribeirão Preto Medical School, University of São Paulo, Ribeirão Preto, Brazil; 2 Department of Oncology, Hospital Imaculada Conceição da Sociedade Portuguesa de Beneficência, Ribeirão Preto, São Paulo, Brazil

**Keywords:** Squamous Cell carcinoma, skin neoplasms, neglected diseases

## Image in medicine

A 37-year-old male patient was admitted at the emergency room with headache, severe pain and bleeding in the tumor. He had a previous diagnosis of squamous-cell carcinoma of the lower lip two years before, however, despite having been informed of the need to excise the initial lesion, he refused treatment due to religious beliefs. The patient returned to seek treatment after 18 months of diagnosis, however the tumor already had great extent, being unresectable. The lesion did not respond to chemotherapy. On examination, the tumor affected all of the right hemiface, with ocular and nasal destruction; jaw and tongue exposure and severe bleeding were present. Unfortunately the patient died 12 days after the hospital admission. Although informed consent allows patients to make treatment decisions based on the most information possible and patient's right to determine his or her treatment, there are a small number of adherents to religious faiths that proscribe conventional care. Despite the efforts for early diagnosis, patients continue to neglect seeking timely treatment. In our case, this was directly responsible for the patient's poor prognosis.

**Figure 1:**
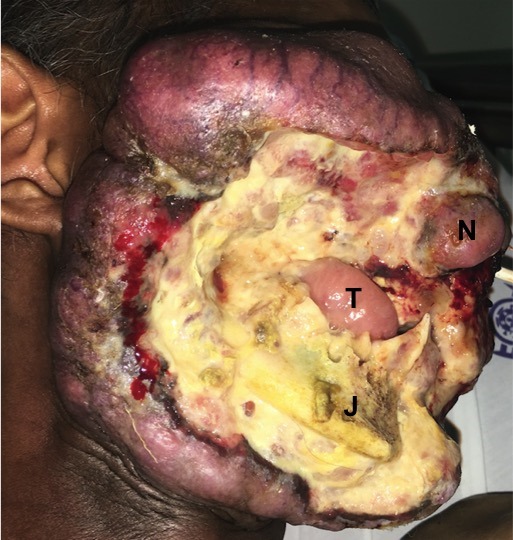
Large invasive squamous cell carcinoma of the lower lip

